# Genomic Introgression in the Hybrid zones at the Margins of the Species' Range Between Ecologically Distinct *Rubus* Species

**DOI:** 10.1002/ece3.70476

**Published:** 2024-11-21

**Authors:** Makiko Mimura, Zhenxing Tang, Shuji Shigenobu, Katsushi Yamaguchi, Tetsukazu Yahara

**Affiliations:** ^1^ Department of Biology Okayama University Okayama Japan; ^2^ Trans‐Omics Facility National Institute of Basic Biology Okazaki Japan; ^3^ Kyushu Open University Fukuoka Japan

**Keywords:** adaptive introgression, climate change, hybrid zone, secondary contact

## Abstract

Populations in extreme environments at the margins of a species' range are often the most vulnerable to climate change, but they may also experience novel evolutionary processes, such as secondary contact and hybridization with their relatives. The range overlap resulting from secondary contact with related species that have adapted to different climatic zones may act as corridors for adaptive introgression. To test this hypothesis, we examined the hybrid zones along the altitude of two closely related *Rubus* species, one temperate and the other subtropical species, at their southern and northern limits on Yakushima Island, Japan. Genomic cline analysis revealed non‐neutral introgression throughout the genome in both directions in the two species. Some of these genomic regions involve gene ontology terms related to the regulation of several biological processes. Our niche modeling suggests that, assuming niche conservatism, the temperate species are likely to lose their suitable habitat, and the backcrossed hybrids with the subtropical species are already expanding upslope on the island. Adaptive introgression through the hybrid zone may contribute to the persistence and expansion of the species in the southernmost and northernmost populations.

## Graphical abstract

Populations in extreme environments at the margins of a species' range are often the most vulnerable to climate change. This study focused on the role of interspecific hybridization at the margins of the species' range of two *Rubus* species (a) and found genomic introgression with non‐neutral transition rates along the hybrids (b).

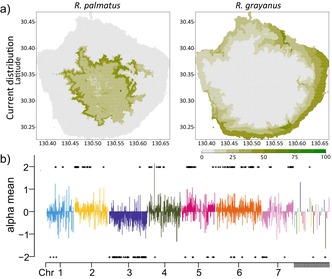

## 1 Introduction

Understanding when an organism will likely persist or adapt in response to environmental change is critical for estimating its impact on biodiversity. In the face of climate change, some species may be at risk of local extinction due to global climate change (Allendorf et al. 2001; Hughes, Daily, and Ehrlich 1997), which ultimately leads to species extinction (Thomas et al. 2004; Urban 2015), whereas other species may cope with these changes by dispersal to more favorable environments, adaptive plastic responses, or adaptation to the changing environment. However, dispersal alone may be insufficient, and niche shifts to tolerate warmer climates may be necessary to keep pace with climate change (Román‐Palacios and Wiens 2020). Long‐term survival and reproduction would likely require organisms to adapt to changing environmental conditions, at least to some extent.

Responses to changes in natural selection require genetic variation either in the form of standing variation or de novo mutations. Although theoretical and empirical studies suggest that recovery from population decline can occur through newly emerging mutations (Bell and Gonzalez 2009; Gonzalez and Bell 2013; Harrison and Larson 2014), rapid adaptation is more likely to occur through standing variation (Orr and Unckless 2014). Standing variations within species may not be solely the result of mutations within species but may also stem from hybridization with related species. In plants, hybridization is common (Mallet 2005; Whitham, Morrow, and Potts 1991), and most species boundaries between related species are semipermeable (Harrison and Larson 2014). Although hybrids in early generations may have lower fitness on average than their parents (Arnold et al. 1999; Barton and Hewitt 1985), ones in later generations have a range of consequences, and some may resolve unfavorable parental allelic associations (Johansen‐Morris and Latta 2006). Therefore, hybridization has sometimes been considered to have positive consequences, as it serves as a source of genetic variation in plants and animals (Goulet, Roda, and Hopkins 2017; Hamilton and Miller 2016; Mallet 2005; Rius and Darling 2014), including adaptive introgression in subsequent generations (Abbott et al. 2013; Suarez‐Gonzalez, Lexer, and Cronk 2018). Recent genomic approaches have further identified the genes or genomic regions involved in adaptive introgression (e.g., Edelman et al. 2019; Ma et al. 2019; Rendón‐Anaya et al. 2021). Considering that the same genomic regions between the relatively distinct lineages in conifer species can be involved in adapting to similar climatic conditions (Yeaman et al. 2016), alleles from related species that are already adapted to certain conditions can be a significant source of potentially beneficial standing variations in focal species.

The positive consequences of hybridization can be more pronounced at the southern and northern edges of the species' range under climate change. These populations at the margins of the species' range are likely to confront the most severe environmental fluctuations within the species' niche; however, they are also expected to have lower within‐population diversity. Demographic and historical effects are expected to decrease genetic variation within peripheral populations, particularly in northern and southern populations as migration tips and tails, respectively (Eckert, Samis, and Lougheed 2008; Hampe and Petit 2005; López‐Delgado and Meirmans 2022). Indeed, gene flow from populations in warmer climates improved the fitness of the northern range margin in *Clarkia pulchella* (Bontrager and Angert 2019). Interspecific hybridization may also provide novel alleles at range margins (Pfennig, Kelly, and Pierce 2016), as secondary contacts among related but ecologically distinct species may be facilitated by climate change (e.g., Fu et al. 2022; Garroway et al. 2010; Mimura et al. 2014). Thus, hybridization and its potential positive and negative consequences may have greater evolutionary significance in peripheral populations of species range.

*Rubus palmatus* Thunb., a temperate raspberry species, and *R*. *grayanus* Maxim., a subtropical species, are in the same sister group but occupy ecologically distinct climate zones (Kikuchi et al. 2022; Okada et al. 2020). The divergence between *R*. *palmatus* and *R grayanus* was relatively recent (approximately 1 Mya), and the latest secondary contact occurred approximately 8000 years ago on Yakushima (Yaku) Island due to climate change, as estimated by coalescent estimations and niche modeling (Mimura et al. 2014). Currently, both *R*. *palmatus* and *R*. *grayanus* have southernmost and northernmost populations on the island, where they are found at higher (1000–1200 m) and lower (50–200 m) altitudes, respectively. Because intrinsic postzygotic isolation would be absent among recently diverged species (Coyne and Orr 2004), these two closely related *Rubus* species hybridize and generate hybrid zones along intermediate altitudinal gradients (200–900 m) on Yakushima Island (Mimura et al. 2014). The hybrid offspring were fertile, and the hybrid zones comprised subsequent generations of hybrids, including *F*_N_ generations and backcrosses (Mimura et al. 2014).

Altitudinal gradients generally show steep environmental changes in abiotic features such as temperature, moisture, and solar radiation, which occur over relatively short distances (Abbott and Brennan 2014; Körner 2007) and may act as selective pressures for the hybrids and parental species. Thus, adaptive traits may be shaped along hybrid zones, even against gene flow between species. For instance, leaf shape, which is a divergent trait between the parental species, showed a sharp and narrow transitional cline along the hybrid zones compared to neutral molecular markers (Mimura and Suga 2020). Although an agent for the selection of leaf shape is currently unknown, these results suggest that divergent selection acted on hybrids against gene flow, resulting in unfavorable alleles moving slower than neutral expectations in the *Rubus* hybrid zones. On the other hand, other alleles might move faster than neutral expectations due to the selection of favoring alleles. Hybridization at the species boundary between temperate and subtropical raspberry species can provide an opportunity to study how hybridization plays a role in the processes of adaptation in response to environmental changes.

In this study, we investigated whether hybrid zones at the southern and northern species range boundaries of *R*. *palmatus* and *R*. *grayanus* can act as corridors for introgression of beneficial alleles. Our hypothesis was that some genes under natural selection might move faster in hybrid zones than neutral expectations toward the parental species. This may ultimately lead to adaptive introgression between the ecologically distinct species in changing environments. To test this hypothesis, we examined the genomic clines of each allele in the hybrid zones and modeled changes in species distribution with climate change.

## 2 Materials and Methods

### 2.1 Sample Sites at the Range Peripheries

**FIGURE 1:**
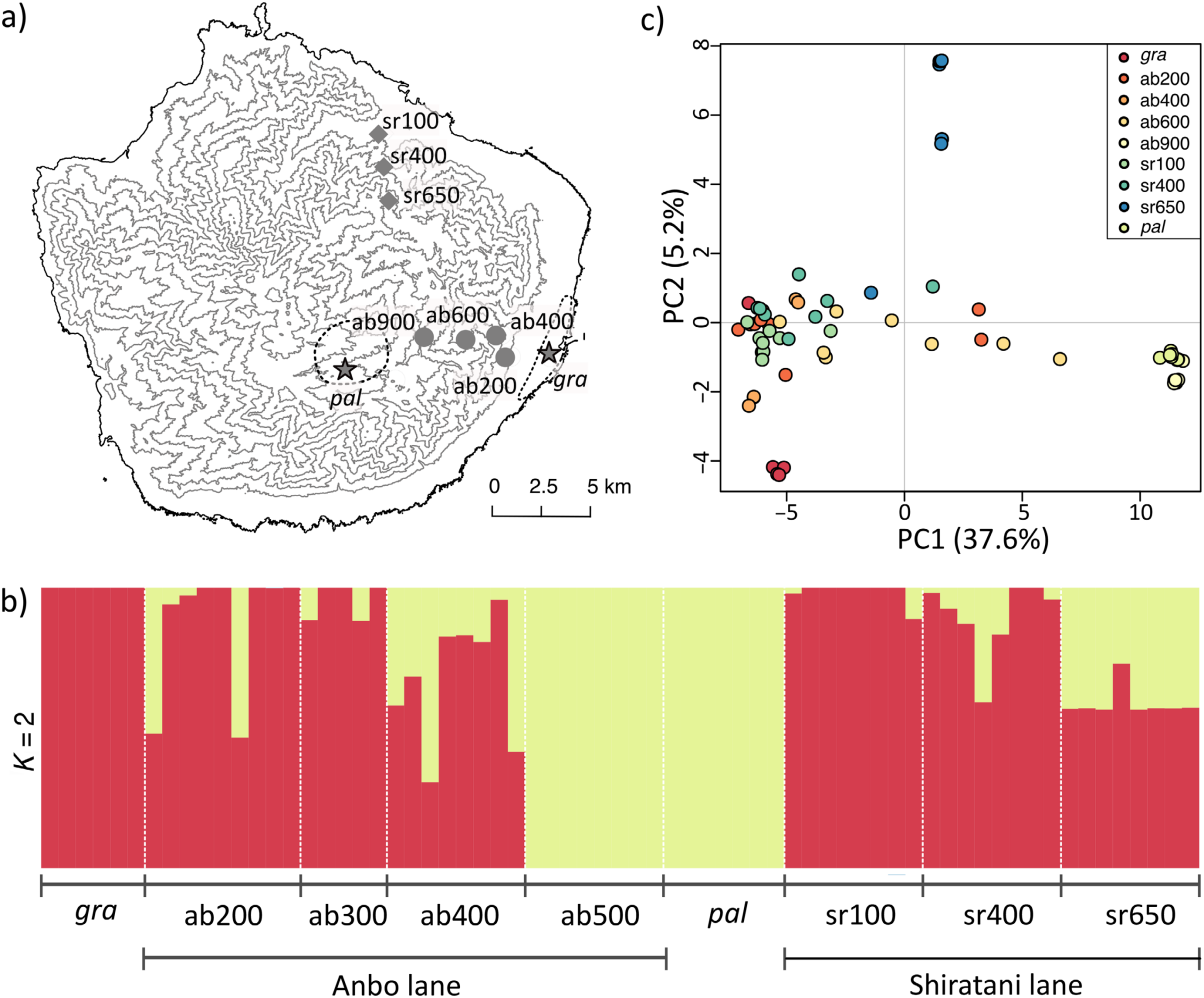
Population structure of the southernmost and northernmost populations of *R*. *palmatus* and *R*. *grayanu*s, respectively, and their hybrid zones on Yakushima Island. (a) Yakushima Island, where two temperate and subtropical species (*R*. *palmatus* and *R*. *grayanus*, respectively) are paratactically distributed. Stars indicate parental populations and dots indicate hybrid populations. Each line represents a contour line every 200 m. (b) ADMIXTURE analysis, and (c) PCA with the LD‐pruned genomic data. *K* = 2 was applied, assuming two sources of gene pools for admixture analysis. The population IDs are shown in Table 1.

*Rubus palmatus* and *R*. *grayanus* are diploid (Thompson 1997) and belong to the subgenus *Idaeobatus*, which rarely shows apomixis (Spies and du Plessis 1986). Our preliminary crossing tests also suggested that they are both self‐incompatible. *R*. *palmatus* is endemic to temperate Japan, while *R*. *grayanus* has a strip distribution from the southern islands of Japan to Taiwan and southern China. Owing to their distinct ecological niches, the current contact zones between these two shrub species are limited to Yakushima Island, which boasts a diverse range of climate zones from cool temperate to subtropical. We collected 21 reference samples as source populations from the southernmost and northernmost populations of *R*. *palmatus* and *R*. *grayanus* on Yakushima Island, respectively. In addition, 54 putative hybrids were collected from the hybrid zones located at approximately 200–900 m elevation along the Anbo (ab) and Shiratani (sr) lanes on the island (Table 1 and Figure 1a). In our previous studies, we identified these pure populations and hybrid zones based on ecological niche modeling, population genetics analysis, and morphological analysis (Mimura et al. 2014; Mimura and Suga 2020). According to these studies, ancestral introgression was also observed in populations outside Yakushima Island; thus, for the sake of simplicity, we used the populations located on Yakushima Island only as the parental populations for the current hybrid zones.

**TABLE 1:** Population sample locations on Yakushima Island and summary statistics. S_N_, number of segregation sites; π, nucleotide diversity. The population IDs starting with ‘ab’ represent the populations along Anbo lane and with ‘sr’ along Shiranani lane.

Population	Pop ID	Altitude	*n*	S_N_	π	Tajima's D	Fu and Li's F	Fu and Li's D
*R*. *grayanus*	gra	45	10	52	0.00055	0.819	1.609	1.615
*R*. *palmatus*	pal	1100	11	42	0.00049	1.372	1.445	1.156
Hybrid zone								
Anbo	ab200	200	9	120	0.00084	−1.063	−1.248	−1.273
	ab400	400	5	108	0.00094	−0.697	−0.620	−0.685
	ab600	600	8	129	0.00140	0.717	1.279	1.230
	ab900	900	8	49	0.00065	1.777	1.638	1.250
Shiratani	sr100	100	8	65	0.00048	−0.919	−1.165	−1.226
	sr400	400	8	112	0.00091	−0.538	−0.237	−0.192
	sr650	650	8	103	0.00135	1.735	1.552	1.150

### 2.2 Draft Genome of *Rubus palmatus* var. *yakumontanus*

Two short and long genomic libraries were constructed for the de novo assembly of *R*. *palmatus* var. *yakumontanus* (“pal03”). The paired‐end library was designed to be 180 bp long, and the insertion size of the mate‐pair library was approximately 3 kbp using the Paired‐End DNA Sample Prep Kit and the Nextera Mate Pair Library Prep Kit, respectively (Illumina, Inc.). Paired‐end and mate‐pair libraries were run on Illumina HiSeq 2000 (101PE) and HiSeq 2500 (101PE), respectively. Raw read data were trimmed using fastq‐mcf with a quality threshold of 20 (PHRED33), a minimum remaining sequence length of 15 bp, and other default settings. The remaining reads were assembled using Platanus (Kajitani et al. 2014), which is a de novo assembler specifically designed for highly heterozygous genomes that assembles short reads into contigs by constructing de Bruijn graphs while optimizing *k*‐mer size, followed by scaffolding of the contigs based on paired‐end information. There were 6302 gap‐closing scaffolds of at least 1000 bp. The N50 scaffold was 212 kbp, and the total length of the scaffolds was 212.9 Mbp. These scaffolds were ordered into chromosome scales using RaGOO (Alonge et al. 2019), which was reference‐guided by the *R*. *occidentalis* L. whole‐genome v3 (VanBuren et al. 2018). *R*. *occidentalis* has same chromosome number as *R*. *palmatus* and *R*. *grayanus* (2*n* = 14). Seven chromosome‐scale scaffolds (206.9 Mb) were aligned. The 3711 unaligned scaffolds had a total size of 5.69 Mbp and an average length of 1533 bp.

RNA sequencing of *R*. *palmatus* var. *yakumontanus* and *R*. *grayanus* was also performed to annotate the draft genome. RNA was extracted from the winter buds, fresh leaves, and flowers of *R*. *palmatus* and *R*. *grayanus*. RNA‐seq libraries were prepared using the TruSeq RNA Library Prep Kit (Illumina, Inc.) and sequenced on an Illumina HiSeq2500 (101PE). The RNA‐seq reads were mapped to the draft genome using TopHat (Trapnell, Pachter, and Salzberg 2009). We then mapped them to the protein sequences of *Fragaria vesca* (Shulaev et al. 2011) and the *Arabidopsis thaliana* database (TAIR database; https://www.arabidopsis.org/) to the pal03 assembly using exonerate (Slater and Birney 2005) and mapp2g (https://github.com/shujishigenobu/mapp2g). Benchmarking Universal Single‐Copy Orthologs (BUSCO version 5.4.4) was used to evaluate the draft genome using the BUSCO dataset embryophyte_odb10 (Simão et al. 2015). The coverage of the core genes was 98.3% (complete and single‐copy BUSCOs: 96.6%; complete and duplicated BUSCOs: 1.7%), indicating that the de novo assembled genome sequences covered most of the genes. We used this draft genome as a reference for genome‐wide SNP detection.

### 2.3 Library Preparation and Variant Call for Hybrid Zones

DNA was extracted using the QIAGEN DNeasy Plant Kit. All DNA samples were quantified using Qubit 2.0 (Thermo Fisher Scientific, Inc.) and used to prepare the library. Double‐digested restriction site‐associated DNA sequence (ddRAD‐seq) libraries were prepared according to protocols developed by Peterson et al. (2012). Briefly, the DNA samples were first digested with two restriction enzymes, EcoRI and MseI, which produce common and rare cut sites in the genome, respectively. This method was designed to increase the number of SNP calls across the samples by excluding rare variants. For each sample, two adaptors were added to the digested samples: a barcode adaptor containing a unique barcode and a common adaptor (Illumina, Inc.). After adaptor ligation, the samples were pooled into two libraries and subjected to polymerase chain reaction using two primers (P1 and P2; Illumina, Inc.). One library of pilot samples was sequenced on an Illumina MiSeq (150PE) lane, and the other libraries were sequenced on an Illumina HiSeq 1500 (101PE) at the National Institute of Basic Biology (Okazaki, Japan).

The generated data were checked for quality using FastQC (https://github.com/s‐andrews/FastQC), filtered by a quality score of more than 20 (PHRED33), and all ambiguous calls (Ns) were omitted. The data were separated into individual datasets with unique barcodes. The individual reads were mapped to the draft sequence data obtained as described above using BWA‐mem (Li and Durbin 2009) with default settings. The mapped reads were then stacked to detect variants with at least ×10 coverage in at least 70% of the samples in each group (gra, pal, and hybrids) using stacks (Rochette, Rivera‐Colón, and Catchen 2019) and vcftools (Danecek et al. 2011). The SNPs were further filtered with a minimum allele frequency of 0.05 and maximum reads of 300, and we then used only the biallelic variants. A total of 32,263 SNPs with an average coverage of 78.9 on chromosome‐scale scaffolds and other small scaffolds (1000 ~ kb) were obtained and used for the following analysis.

### 2.4 Genome‐Wide Genetic Differentiation

We inferred genome‐wide population structure and differentiation along the hybrid zones of *R*. *palmatus* var. *yakumontanus* and *R*. *grayanus*. Because these analyses do not assume linked loci, SNPs were pruned based on linkage disequilibrium (LD) values using sliding windows of 50 SNPs with a spacing of 10 SNPs, and we retained only those with *R* ^*2*^ values ≤ 0.2. The retained 1163 SNPs were used for ADMIXTURE (Alexander, Novembre, and Lange 2009) and principal component analysis (PCA). ADMIXTURE was used to estimate the maximum likelihood of individual ancestries. We assumed *K* = 2 to evaluate the genetic components of the hybrid zones with the two parental sources, *R*. *palmatus* var. *yakumontanus* and *R*. *grayanus*. PCA was also performed to illustrate the variations in the hybrid zones.

### 2.5 Detecting Outlier SNPs

The Bayesian genomic cline model with genotype uncertainty (Gompert and Buerkle 2011, 2012) implemented in the *bgc* program (Gompert and Buerkle 2012) was used to quantify introgression of each SNP along the hybrid index. This model quantifies locus‐specific ancestry as a function of genome‐wide admixture, the hybrid index of individuals (here, the probability of *R*. *palmatus* ancestry), with two parameters (*α* and *β*) based on Bayesian estimation. Parameter *α* denotes a cline center and changes in the probability of ancestry from one species to another, and *β* determines the rate of transition. A positive *α* value indicates that an allele specific to *R*. *palmatus* var. *yakumontanus* is likely to be found in the hybrids even when the hybrid index becomes lower (hybrids backcrossed with *R*. *grayanus*) and *vice versa* when *α* is negative. Parameter *β* indicates the rate of transition, which means that a positive *β* has a sharp transition along the hybrid index gradient, while a negative *β* indicates a slow rate of transition and an increased likelihood that heterozygotes will be found in the intermediate hybrid index.

We selected ancestry‐informative loci (AILs) for genome cline analysis from the entire SNP dataset. Loci that were nearly fixed between the parental populations (*F*_ST_ ≥ 0.9) were selected as AILs, resulting in 10,145 SNPs. The read coverage of each allele of the AILs was used as input data to account for genotype uncertainty (number of reads per allele), and sequencing errors and linkages between loci were also considered to model the introgression. The estimation of the genomic cline models was performed with 50,000 MCMC sampling every 10th step and after a 25,000‐step burn‐in. Significant loci were identified with parameter values for which the upper and lower 99% confidence intervals of each estimated parameter did not cross zero.

In addition to genomic cline analysis, outliers were tested based on the PCA of a scaled genotype matrix using the R package *pcadapt* (Privé et al. 2020) to confirm the loci associated with adaptive divergence between the parental species. The program assumes that SNPs that are excessively related to the population structure are candidates, in our case, for species divergence. The genotypes of each parental population were first phased using Beagle version 5.4 (Browning et al. 2021). Phased genotypes with minor allele frequencies of more than 0.05 (resulting in 33,221 SNPs) were used to detect outliers, assuming *K* = 2. SNPs with FDR‐adjusted *q*‐values less than 0.01 were defined as outliers.

### 2.6 Annotation and GO Enrichment of Candidate Genomic Regions

We identified the closest upstream and downstream putative genes within 5 kb of the candidate SNPs identified using both genomic cline analysis and *pcadapt*. Putative functional genes in the vicinity of significant SNPs were annotated with *A*. *thaliana* reference protein sequences using bedtools (Quinlan and Hall 2010). The first closest genes within or near the candidate SNPs are listed. GO enrichment analysis for genes that were physically close to SNPs with significant cline parameters was performed using PlantRegMap (Tian et al. 2020). A list of putative homologs located in or near all SNPs detected in this study was used as the background, and each of the four datasets of significant SNPs (i.e., SNPs with positive or negative *α* or *β*) were used as queries.

### 2.7 Ecological Niche Modeling

Ecological niche modeling was used to estimate the current and future potential distribution ranges of *R*. *palmatus* var. *yakumontanus* and *R*. *grayanus* on Yakushima Island, representing the southern and northern limits of *R*. *palmatus* and *R*. *grayanus*, respectively. Data on the presence or absence of these species on Yakushima Island between 2000 and 2005 were obtained from 238 sites on the island (Yahara et al., unpublished). We added our own observation sites of the parental species, resulting in a total of 244 sites. Among the sites observed, *R*. *palmatus* var. *yakumontanus* and *R*. *grayanus* were recorded at 28 and 33 sites, respectively. The following five climate variables (averages between 1960 and 1990) were obtained from a high‐resolution climate database for the Asia‐Pacific region, ClimateAP (Wang et al. 2019), and used for niche modeling: mean annual temperature (MAT), temperature difference between the mean warmest month and mean coldest month (TD), mean annual precipitation (MAP), and annual heat‐moisture index (AHM). These climate variables were selected because they were relatively less correlated with each other on the island (mean absolute Pearson pairwise correlation of 0.54). We ensembled the predictions of five models: GLM, MARS, FDA, RF, and Maxent; each model was run in 10 replicates, and the mean of all models was used as a predicted range of potential distributions. All predictions and ensembles were performed using the R package, BIOMOD2 (Thuiller 2003). Future distributions were projected using the future climate model CCSM4 rcp45 for the year 2085 obtained from ClimateAP (Wang et al. 2019).

### 2.8 Observation of Flowering Time

The flowering time was observed at different altitudes to investigate the overlapping flowering periods for hybridization. The proportion of flowering plants was observed in two parental species and four hybrid populations at the Anbo lane on Yakushima Island, approximately every 2 weeks from February to May 2018. On average, 18 individuals per population were observed. Individuals from the island were also observed in the common garden, as described in Mimura and Suga (2020). Days to first flowering were observed for eight *R*. *grayanus* individuals, 11 hybrid individuals (eight from ab600 and three from sr600), and 11 *R*. *grayanus* individuals once every 2 or 3 days a week in the spring of 2019.

## 3 Results

### 3.1 Population Genomic Structure in the Hybrid Zones

The hybrid populations had higher nucleotide diversity (*π* = 6.5–14.0 × 10^−4^) compared to the two parental populations (π = 4.9–5.5 × 10^−4^; Table 1). The SNP dataset without high LD values showed gradual variation in genomic composition along the altitudinal gradient (Figure 1). ADMIXTURE generally showed asymmetric hybridization (Figure 1b and Figure S1). The hybrids were more likely to be backcrossed with *R*. *grayanus* than with *R*. *palmatus*. At intermediate altitudes (i.e., 600–650 m in this study), most of the individuals were hybrids. PCA showed a similar trend (Figure 1c), with the first (37.6%) and second (5.2%) components explaining most of the variation. The parental populations of *R*. *palmatus* and *R*. *grayanus* diverged, based on the SNP set (global *F*_ST_ = 0.679).

### 3.2 Genomic Cline Analysis and Signatures of Introgression

**FIGURE 2:**
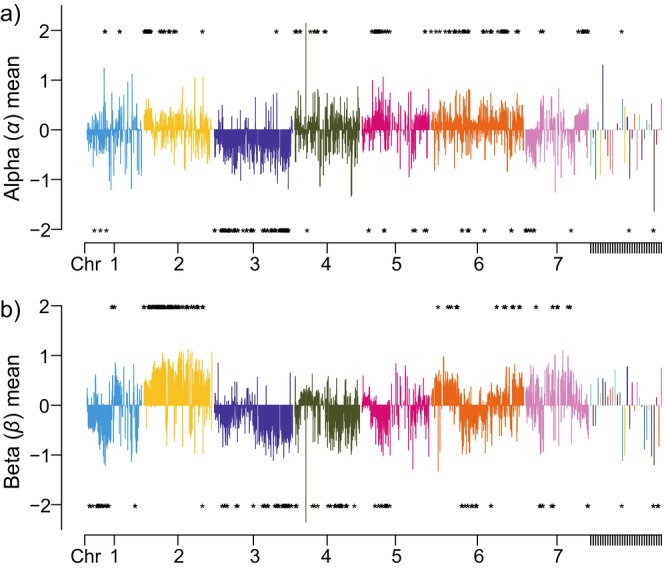
Cline parameters of the Bayesian genomic cline analysis alpha values (a) and beta values (b). Alpha values indicate the center of the cline, and beta values indicate the slope of the cline. Asterisks indicate the sites detected by both genomic cline analysis and *pcadapt*.

**FIGURE 3:**
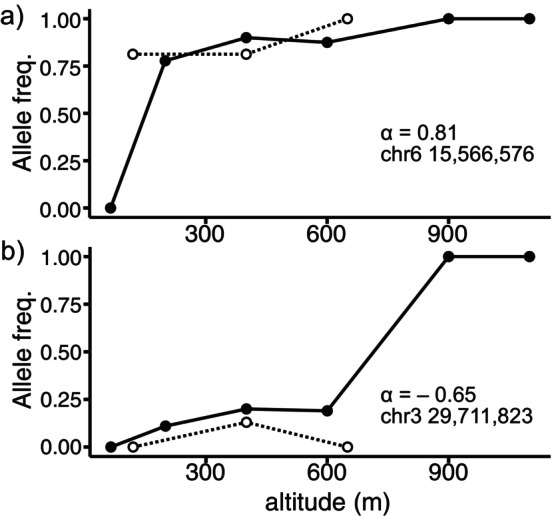
Genomic clines of the alleles with significant alpha and negative beta along the altitude gradient. Allele frequencies of *R*. *palmatus* alleles were calculated as population means (i.e., 1.0 means that the *R*. *palmatus* allele was fixed) and plotted on the y‐axis. Solid lines and dashed lines represent altitudinal transect lines, the Anbo and Shiratani lanes, respectively. (a) An allele with a positive alpha on chromosome 6 located within a transcriptome putatively homologous to SRD2. (b) An allele with negative alpha on chromosome 3 located closest to a transcriptome putatively homologous to AGAMOUS. Both were also detected by *pcadapt* as significant divergences between the parental species.

Genomic cline analysis was performed using AILs with two parental populations (i.e., gra and pal populations) as reference populations, and hybrid indices were calculated for the individuals collected along the hybrid zones. Estimates of the genomic cline parameters *α* and *β* of each AIL against the hybrid index were highly variable among loci. We identified 2219 significant loci throughout the genome (Figure 2 and Table S1) and found 518 AILs with excess *R*. *palmatus* ancestry with significant positive *α* values. Some of the detected AILs were closely linked and were located near or within 129 putative autosomal transcriptomes. Conversely, 714 AILs had significantly negative *α* values, indicating excess *R*. *grayanus* ancestry along the hybrid index. These AILs were located near or within the 189 putative transcripts. We also identified 698 AILs with significantly positive *β* values, indicating a steep transition in genomic cline. These AILs were located near or within 132 putative transcripts. Finally, 530 AILs with significantly negative β values were detected, which were in or close to 135 putative functional genes, indicating a reduced rate of genomic cline (i.e., wider cline). Twenty‐five of the AILs with positive *α* and 123 of the AILs with negative *α* also exhibited significant negative *β* values. Of the AILs with significant *α* or *β* values detected by *bgc* analysis, 1827 AILs were also detected by *pcadapt* (Figure 2, Table S1). Some of the genetic regions with high genomic cline parameters were not found in *pcadapt*. The candidate AILs were located in 345 putative transcriptomes. These candidates with positive or negative *α* cline centers exhibited contrasting trends in their transition along altitudes where the hybrid zones were located between the parental species (Figure 3).

We performed GO enrichment analysis to test whether any GO terms were overrepresented in the annotated genomic regions, with significant cline parameters detected by both *bgc* and *pcadapt*, or *bgc* only. The analyses that were applied to each of the four gene sets revealed significant GO terms (Table S2). The significant 15 GO terms for positive *α* loci were detected, including regulation of biological quality (GO:0065008). Significant GO terms for negative *α* loci included cellular response to osmotic stress (GO:0071470), and 10 other GO terms. For *β* loci, 23 GO terms, including male gamete generation (GO:0048232), and six GO terms were found in the positive and negative *β* gene datasets, respectively. GO enrichment analysis of the dataset comprising alleles detected by *bgc* only showed somewhat consistent results (Table S3).

### 3.3 Ecological Niche Modeling

**FIGURE 4:**
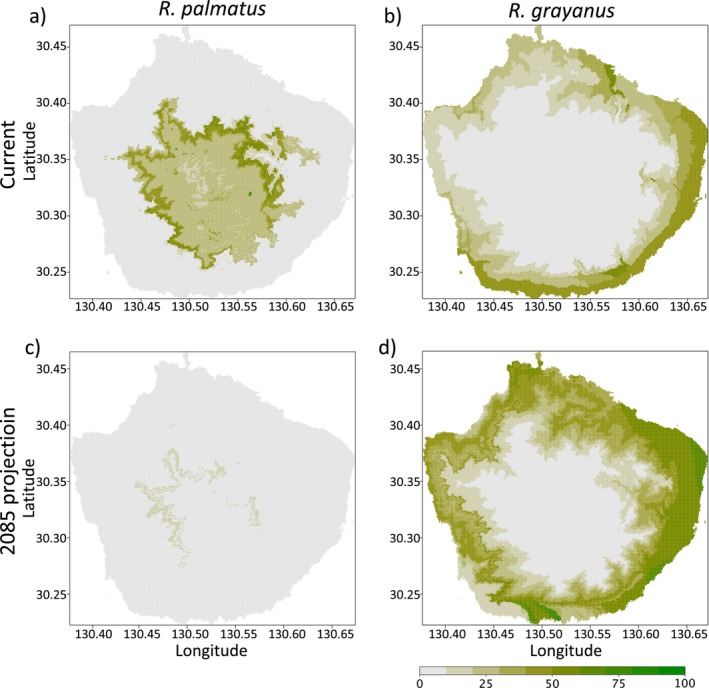
Ensemble niche projections based on past climate data (1960–1990) and climate model CCSM4 rcp4.5 for 2085. Four relatively uncorrelated climate datasets (MAT, TD, MAP, and AHM) were used for modeling. The probability of occurrence was calculated as the mean of six different models (GLM, GAM, GBS, RF, CTA, and MAXENT) based on the presence/absence data from 238 locations on the island for *R*. *palmatus* (a) and *R*. *grayanus* (b). The CCSM4 rcp45 climate model was used for the 2085 projection (c, d).

Based on the average climate data for 1960–1990, the current distributions predicted and ensembled by the models indicated that *R*. *palmatus* var. *yakumontanus* is expected to occur in mountainous areas at higher elevations than *R*. *grayanus* on Yakushima Island (Figure 4a,b). The estimated distribution areas with a probability of at least 50% occurred at approximately 738–1514 m asl for *R*. *palmatus* var. *yakumontanus* and 0–336 m asl for *R*. *grayanus*. The projection based on a future climate scenario indicated that *R*. *grayanus* populations are likely to expand, whereas areas with suitable environments for *R*. *palmatus* var. *yakumontanus* are expected to decrease on the island (Figure 4c,d).

### 3.4 Flowering Time and Reproductive Isolation

**FIGURE 5:**
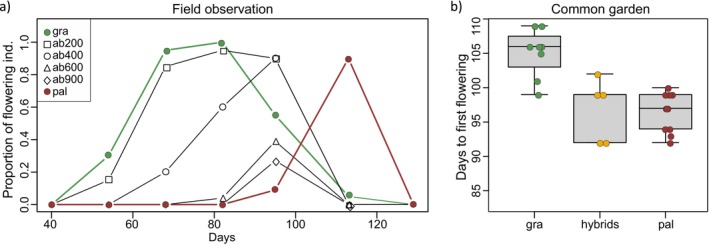
Flowering time and first flowering date of the parental and hybrid populations in the (a) island and (b) common garden experiments, respectively. Proportions for flowering plants were estimated from 20 m transects at each observed altitude on the island every other week in 2018. The first flowering date was observed every other day in 2019.

Flowering began with the *R*. *grayanus* population in early spring in the lowlands of the island. The flowering peaks of *R*. *grayanus* and *R*. *palmatus* in their natural distribution were approximately 80 and 115 Julian days, respectively, in 2018 (Figure 5a). The flowering period of *R*. *grayanus* was longer than that of *R*. *palmatus*; therefore, the end of the flowering period of *R*. *grayanus* overlapped with the beginning of the flowering period of *R*. *palmatus*. The flowering time of the hybrid populations was intermediate to that of the parental populations on the island. In the common garden experiment, the trends in flowering time were reversed, with *R*. *palmatus* flowering earlier than *R*. *grayanus* (Figure 5b). The maximum difference in the first flowering date between the species was 17 days. The flowering times of the hybrids were similar to those of *R*. *palmatus*.

## 4 Discussion

### 4.1 Changes in Species' Ranges on the Island and Secondary Contact

The southernmost and northernmost populations often experience extreme environments within the species' range and could be the most vulnerable to climate change. Our ecological niche modeling suggested that the peripheral populations of *R*. *palmatus* and *R*. *grayanus* have distinct ecological niches on the island. Under a moderate climate warming scenario, the southernmost populations of *R*. *palmatus*, distributed at higher altitudes on the island, would lose most of their suitable habitats, whereas its lowland congener *R*. *grayanus* would expand its range upslope. Rapid upslope migration due to climate change has been observed in many plant species (Chen et al. 2011; Gottfried et al. 2012). In this study, our genomic data suggest that the projected area of *R*. *grayanus* range expansion in the lowlands is currently occupied mostly by hybrids and backcrossed individuals toward *R*. *grayanus*. This implies that the northernmost population of *R*. *grayanus* may have already benefited from introgression from *R*. *palamatus* at intermediate altitudes. Conversely, *R*. *palmatus* faces potential habitat loss at its southernmost population of the species' range, as climate change‐induced range shifts expect habitat loss at the warm edge (lower latitudes and elevations) of the species' ranges (Wiens 2016). Since the mutation–area relationship follows a power law (Exposito‐Alonso et al. 2022), it would be challenging for *R*. *palmatus* to persist on the island because of habitat loss and reduced genetic variation if the niche of the species is presumed to remain constant.

### 4.2 Asymmetric Introgression in the Hybrid Zones

Interspecific hybridization is often asymmetrical in plants (Abbott 2017; de Lafontaine and Bousquet 2017). Many hybrid progenies were genetically more similar to *R*. *grayanus*, indicating asymmetric introgression with a higher frequency of backcrossing with *R*. *grayanus* than with *R*. *palmatus*, especially at lower altitudes. When F1 hybrids are fertile, they are likely to mate with more abundant species at mating time than with rarer species and generate asymmetric gene flow (Burgess et al. 2005; Lepais et al. 2009). Thus, overall neutral asymmetric introgression can potentially be formed by differences in flowering abundance. Our field observations indicated that the flowering of *R*. *grayanus* at lower altitudes was more abundant and extended than that of *R*. *palmatus*, potentially resulting in an asymmetric gene flow. Alternatively, but not exclusively, the differences in population dynamics affect range shifts and population expansion (Canestrelli et al. 2016; Currat et al. 2008; Guichoux et al. 2013). This also predicts more introgression toward a colonizing and expanding species because alleles of residential species that permeate into a colonizing species would rapidly increase in frequency with colonization, while a resident species would already reach its carrying capacity. Thus, *R*. *grayanus* backcrosses carrying alleles of *R*. *palmatus* probably expand toward intermediate altitudes, whereas *R*. *palmatus* backcrosses carrying alleles of *R*. *grayanus* are less abundant.

The asymmetric introgression and genetic population structure of the hybrid zones are generally concordant with our previous study (Mimura et al. 2014; Mimura and Suga 2020); however, the population at the higher hybrid zone in altitude (ab5 in the Anbo lane) showed much less introgression from *R*. *grayanus* than in these previous studies, despite the fact that most individuals used in this study were also used in the previous studies. This inconsistency is probably caused by differences in molecular markers. Our genome‐wide SNPs were randomly representative across the genome, while several gene sequences in the previous studies were selected based on polymorphisms, which may have affected the proportion of foreign alleles in the hybrid zones. This suggests that the individuals in hybrid zones at higher altitudes may include relatively later generations of backcrosses with *R*. *palmatus*.

### 4.3 Non‐Neutral Transition of Introgressive Alleles in Hybrid Zones

Bidirectional introgression between the two parental populations along the hybrid zones was also found in genome cline analysis. Hybridization can have detrimental consequences for a focal population that receives foreign alleles but also facilitates adaptive introgression (Suarez‐Gonzalez, Lexer, and Cronk 2018) and evolutionary rescue (Stelkens et al. 2014) by providing genetic variation, including novel mutations. The hybrid zones we studied were located across the relatively sharp gradient of altitude (approximately 126.1–204.3 m in elevation per kilometer), which is often associated with temperature and other environmental variables that can act as selective pressures to determine species distributions (Abbott and Brennan 2014; Angert and Schemske 2005; Brennan et al. 2009; Kimball and Campbell 2009). Our genomic cline analysis revealed that some AILs spread faster than neutral expectations in both directions along the altitudes, which implies adaptive introgression. Although there would be some false‐positive AILs, these sets of genomic regions also showed significant GO enrichment, suggesting that non‐neutral introgression can be associated with specific functions.

For example, the SNPs that were transferred from *R*. *grayanus* to more *R*. *palmatus*‐like individuals (i.e., negative *α* cline center) included a genomic region potentially associated with phenology. Clines along an environmental gradient of phenological traits and their associated SNPs indicate their adaptive significance. Both *Rubus* species on the island generally begin flowering almost simultaneously with leaf bud flash in early spring, with flowering time shifting from low to high altitudes. However, when both species were placed in a common garden, the flowering time shifted from high to low altitude populations in spring, while the bud flash time shifted from low to high altitude populations, suggesting that the phenology of the parental species has genetic differences in response to environmental cues. Although the detailed environmental cues in these species are still unclear, the flowering time of a raspberry cultivar (*R*. *idaeus*), which belongs to the same subgenus as our study species, was promoted by temperature and long‐day light conditions after several weeks of chilling (Sønsteby and Heide 2008). Therefore, flowering time could potentially be considered an adaptive trait that is influenced by altitudinal gradients, where temperature variations exist but the photoperiodic cycle remains constant. In addition, the set of AILs showing excess *R*. *palmatus* ancestry (positive *α* cline center) showed significant GO enrichment. For example, among the 15 genomic regions involved in the GO related to regulation of biological quality, the putative homolog SRD2 is associated with morphogenesis with temperature sensitivity in *Arabidopsis* (Ohtani and Sugiyama 2005). Some of the candidate AILs with a significant cline center shift (positive or negative *α*) also had a significantly widened cline (negative *β*), indicating that adaptive introgression was promoted by environmental gradients. Although further investigation is required, these findings suggest that they are likely to be genomic regions associated with adaptive introgression.

Divergent loci in the hybrid zones with sharp transitions (positive *β* in cline analysis) would represent genetic regions associated with reproductive isolation. Hybrid populations may avoid speciation breakdown through the divergent selection of intrinsic reproductive loci (Chaturvedi et al. 2020). In this study, several loci with significantly narrow cline width have been identified. For instance, an enriched GO term ‘sexual reproduction’ was detected, and these associated AILs may represent regions of the genome that contribute, at least in part, to interspecific reproductive isolation. No apparent strong intrinsic reproductive isolation was observed between the focal species (Mimura and Suga 2020), but it became stronger with increasing genetic distance in the *Rubus* species (Okada et al. 2020). The function and associated phenotypes of these loci in *Rubus* need to be determined to test whether divergent selection acts on specific genomic regions associated with reproductive isolation. Nevertheless, it was evident that certain genomic segments were transferred across the hybrid zones at non‐neutral rates, utilizing the hybrid zones as a temporarily formed corridor for gene flow during recurrent climatic fluctuations.

Hybrid zones along altitudinal gradients offer valuable insights into the ecological and evolutionary processes that drive species divergence and adaptive introgression (Abbott and Brennan 2014; Körner 2007). This study suggests putative adaptive introgression at the margin of the species' range between closely related but ecologically distinct species due to secondary contacts caused by repeated climate changes. Yakushima Island has the southernmost or northernmost populations of several plant species, probably because of its geographic location and the high mountains on the island. Thus, this island provides an excellent system for examining the adaptive divergence and maintenance of species boundaries. Although our study focused on the current hybrid zones on the island, multiple contacts and introgressions may have occurred over the course of repeated climate change (Mimura et al. 2014). Such species dynamics, with fluctuating environmental changes leading to isolation and contact, could have contributed to the persistence and robustness to environmental changes at the margins of the species range.

## Author Contributions

**Makiko Mimura:** conceptualization (lead), data curation (lead), formal analysis (lead), funding acquisition (lead), investigation (lead), methodology (lead), project administration (lead), resources (equal), visualization (equal), writing – original draft (lead), writing – review and editing (lead). **Zhenxing Tang:** data curation (equal), formal analysis (supporting), writing – review and editing (supporting). **Shuji Shigenobu:** data curation (equal), methodology (equal), writing – review and editing (supporting). **Katsushi Yamaguchi:** data curation (equal), methodology (equal), writing – review and editing (supporting). **Tetsukazu Yahara:** conceptualization (equal), investigation (equal), writing – review and editing (equal).

## Conflicts of Interest

The authors declare no conflicts of interest.

## Supporting information


Data S1.

## Data Availability

All raw sequence reads were deposited in the DDBJ Sequence Read Archive (DRA) and NCBI Sequence Read Archive (SRA). Data registration is in progress (SSUB ID: SSUB025228 for DDBJ).
