# Supplementary material for: Genomic Introgression in the Hybrid zones at the Margins of the Species' Range Between Ecologically Distinct Rubus Species
Source: Ecol Evol. 2024 Nov 21;14(11):e70476. doi: 10.1002/ece3.70476 (PMC11581777; doi:10.1002/ece3.70476)
Supplement: Supplementary file 1 — Data S1. [file ECE3-14-e70476-s001.docx]

**Table S1**. The top 20 loci with significant values for the genomic cline parameters (bgc), which also showed significant divergence between the species by pcadapt. Bold font indicates significant values. LB and UB: lower and upper bounds of 99% confidence interval; putative: annotated gene from the database; distance: bp from the SNP.

**Table S2.** GO enrichment analysis of the putative genes on or near the SNPs with significant values for the genomic cline parameters and significant divergence between the parental species (pcadapt).

**Table S3.** GO enrichment analysis for the putative genes on or near the SNPs with significant values for genomic cline parameters.

**Figure S1**. ADMIXTURE when K=2 or 3. An LD-pruned dataset was used.
